# Targeted Therapy in HER2 Exon 20-Mutant Non-small Cell Lung Cancer With Leptomeningeal Disease: A Case-Based Approach to Treatment Decision-Making

**DOI:** 10.7759/cureus.88905

**Published:** 2025-07-28

**Authors:** Osama Elzaafarany

**Affiliations:** 1 Neuro-Oncology, Moffitt Cancer Center, Tampa, USA

**Keywords:** cns metastases, exon 20 insertion, her2 mutation, leptomeningeal disease, non-small cell lung cancer, precision oncology

## Abstract

A rare and clinically challenging case of leptomeningeal disease (LMD) secondary to HER2-mutated non-small cell lung cancer (NSCLC), marked by both exon 20 insertion and gene amplification, is presented. In the absence of LMD-specific therapeutic guidelines for HER2-driven NSCLC, treatment was initiated with trastuzumab deruxtecan (Enhertu), based on extrapolated evidence from clinical trials in systemic NSCLC and HER2-positive breast cancer with central nervous system involvement. The patient, a 69-year-old woman with good performance status, developed LMD following prior systemic therapy and local CNS treatments. MRI and CSF cytology confirmed the diagnosis. Given the lack of approved therapies for HER2-mutant NSCLC with LMD, the treatment strategy was informed by available clinical data and supporting literature on the CNS activity of Enhertu. Other options such as poziotinib and zongertinib were considered but not pursued. This case underscores the urgent need for inclusive clinical trials addressing CNS complications in molecularly defined NSCLC and demonstrates the real-world application of precision oncology beyond trial populations.

## Introduction

Leptomeningeal disease (LMD) in non-small cell lung cancer (NSCLC) represents a rare and therapeutically challenging complication, particularly when associated with uncommon oncogenic drivers. LMD is observed in approximately 3-9% of NSCLC cases, underscoring its rarity and complexity. Without any therapy, the prognosis for LMD is generally poor, with median survival often measured in weeks. However, medical interventions such as whole-brain radiotherapy (WBRT) and chemotherapy can offer some improvement in survival and quality of life, though their efficacy varies. WBRT may provide symptomatic relief, while chemotherapy's effectiveness is often limited by the blood-brain barrier [1].

Human epidermal growth factor receptor 2 (HER2) alterations, including exon 20 insertions and gene amplification, are detected in a small subset of NSCLC patients, with mutations found in approximately 2-4% and amplification occurring in about 2-5% of cases. The coexistence of both mutations in the context of LMD is exceedingly rare. Treatment guidelines in this molecular and anatomic setting are lacking, especially as clinical trials frequently exclude patients with LMD. Conducting a molecular study of LMD itself could refine therapy by identifying specific targets for treatment, potentially improving outcomes [1].

This case exemplifies the therapeutic dilemma and highlights a rationale for extrapolating systemic data to guide treatment, emphasizing the need for personalized treatment strategies and the importance of expanding clinical trial eligibility to include patients with such complex presentations.

This article was previously posted to the Research Square preprint server on June 17, 2024, under the title “Targeted Therapy Decision in HER2 Exon 20-Mutant Non-small Cell Lung Cancer With Leptomeningeal Disease: A Case-Based Perspective”.

## Case presentation

A 69-year-old female ex-smoker presented in early 2024 with respiratory symptoms and right cervical lymphadenopathy. CT scans revealed a right upper lobe mass with associated mediastinal lymphadenopathy and distant metastases to the brain and bone. Bronchoscopic biopsy from mediastinal lymph nodes confirmed lung adenocarcinoma, staged as T4N2M1c. Immunohistochemistry demonstrated programmed death-ligand 1 (PD-L1) expression <1%. Circulating tumor DNA analysis using the Guardant360 assay detected a HER2 exon 20 insertion mutation, along with HER2 gene amplification.

The patient was initiated on first-line systemic therapy with carboplatin, pemetrexed, and pembrolizumab, followed by maintenance pemetrexed and pembrolizumab. Stereotactic radiosurgery (SRS) was delivered to multiple brain metastases, and palliative external beam radiation therapy was administered to osseous sites of disease.

In April 2025, the patient developed worsening headaches. Brain MRI revealed multifocal leptomeningeal enhancement with stable parenchymal metastases. Lumbar puncture confirmed leptomeningeal metastases (LMD) via positive cerebrospinal fluid (CSF) cytology. Despite this diagnosis, she remained neurologically intact, with non-focal examination findings and no requirement for corticosteroids or antiepileptic medications.

**Figure 1:**
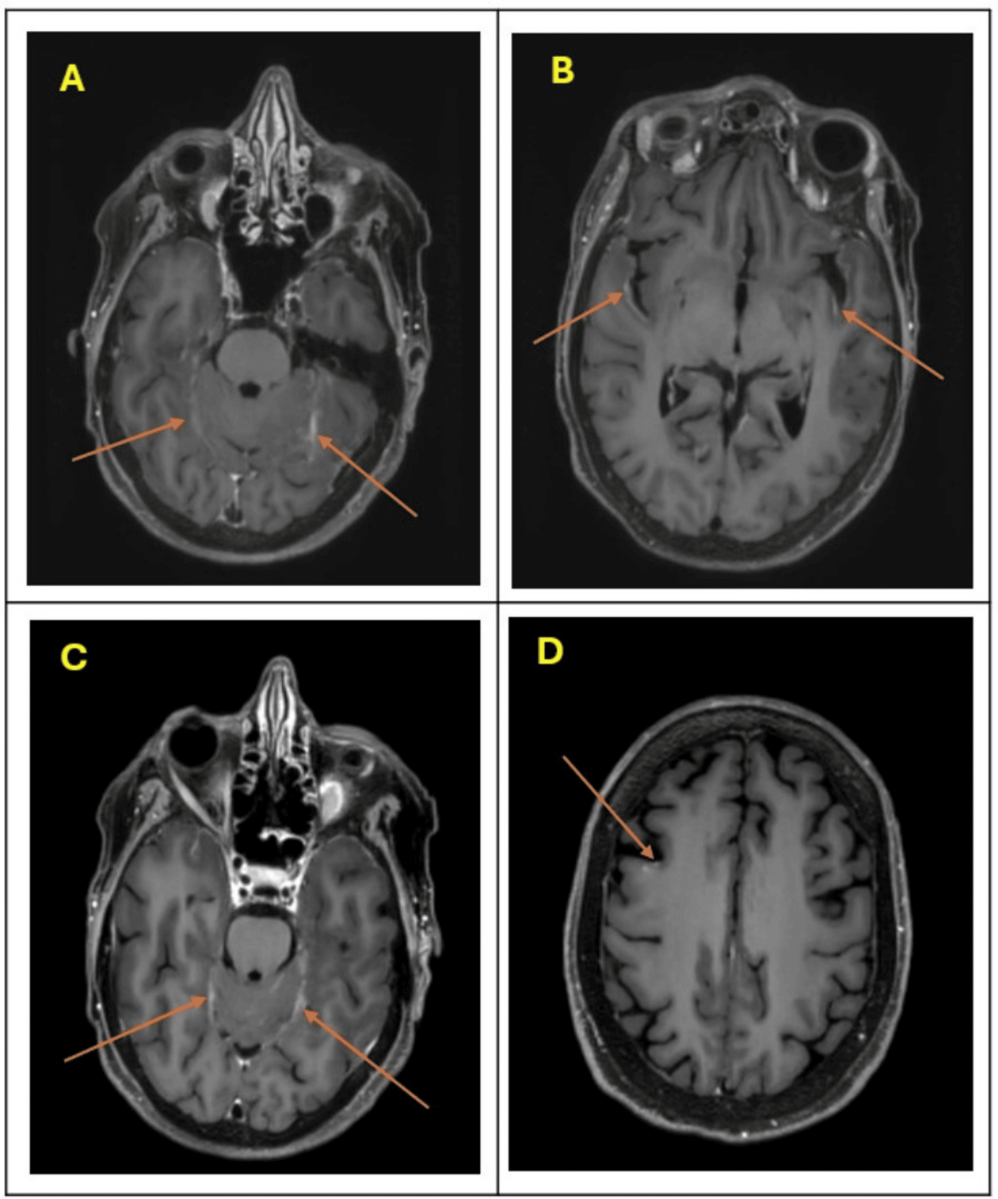
Axial contrast-enhanced brain MRI demonstrating leptomeningeal disease (LMD). Axial post-contrast T1-weighted MRI of the brain (labeled A–D) demonstrating abnormal enhancing lesions (arrows) concerning for leptomeningeal disease (LMD). (A) and (C): Enhancement along the bilateral cerebellar folia and posterior fossa leptomeninges. (B): Leptomeningeal enhancement involving the bilateral temporal lobes. (D): Focal gyral enhancement over the high convexity region, suggestive of cortical involvement. These imaging findings are characteristic of LMD, often seen in advanced metastatic disease.

The case was referred to our institution in May 2025. Repeat lumbar puncture again demonstrated atypical cells in the CSF, with normal glucose and protein levels. MRI findings were consistent with diffuse leptomeningeal involvement (Figure 1). The only presenting neurologic symptom was a persistent headache. Given her preserved performance status, the patient was treated with whole neuraxis proton radiotherapy.

In the absence of standard-of-care therapies for HER2-mutant NSCLC with LMD, and the limited efficacy of currently available tyrosine kinase inhibitors in this molecular subtype, the decision was made to initiate systemic therapy with trastuzumab deruxtecan (Enhertu), a HER2-directed antibody-drug conjugate. At the time of this report, follow-up data following treatment initiation are not yet available.

## Discussion

The use of trastuzumab deruxtecan in this case was informed by several lines of evidence. The DESTINY-Lung02 trial demonstrated the efficacy of this agent in HER2 exon 20-mutant NSCLC, though it excluded patients with LMD [2]. Its CNS activity is better characterized in HER2-positive breast cancer, where studies such as the DEBBRAH trial and ROSET-BM have reported responses in patients with brain metastases and LMD [3,4]. Additional retrospective studies have supported its ability to cross the blood-brain barrier and generate meaningful responses in LMD from breast cancer [5].

Alternative options were considered, including Poziotinib, a pan-HER tyrosine kinase inhibitor. This drug demonstrated clinical benefits in a phase 2 study involving 50 patients with EGFR exon 20 mutant advanced NSCLC [6]. Additionally, a case report by Fan et al. described a clinical response to Poziotinib in a patient with HER2 exon 20-mutated NSCLC with LMD [7]. However, the current evidence is insufficient to support its use specifically for LMD. There are also concerns regarding the risk-benefit ratio and toxicity observed in clinical trials, highlighting the need for further data from randomized controlled studies.

Zongertinib, an emerging irreversible HER2-selective tyrosine kinase inhibitor, has shown promising results in a phase 1 study for previously treated HER2-mutant NSCLC. However, its efficacy in the subset of patients with LMD is lacking [8]. Nevertheless, Zongertinib may be the preferred second-line option when trastuzumab deruxtecan (Enhertu) is contraindicated or not tolerated, particularly in patients experiencing severe fatigue or poor performance status in the setting of whole neuraxis radiotherapy, which is a common practice in managing LMD.

Intrathecal trastuzumab was not pursued, as HER2 exon 20 insertions typically affect the intracellular domain, limiting the utility of extracellular monoclonal antibodies [9,10].

This case underscores the challenge of treating LMD in molecularly defined NSCLC subsets. Given the rarity of HER2 exon 20 insertions with concurrent amplification and LMD, therapeutic decisions must often rely on extrapolated evidence from related disease settings.

## Conclusions

This case highlights a rare molecular and anatomic scenario: HER2 exon 20-mutated and amplified NSCLC with LMD. In the absence of guideline-recommended therapies, we utilized trastuzumab deruxtecan based on extrapolated data from NSCLC and breast cancer trials. This case emphasizes the importance of expanding clinical trial eligibility to include patients with LMD, as well as the need for dedicated studies addressing treatment strategies in this high-risk population.
